# Problems in Audiovisual Filtering for Children with Special Educational Needs

**DOI:** 10.1177/2041669520951816

**Published:** 2020-08-26

**Authors:** Stephanie Armstrong-Gallegos, Roderick I. Nicolson

**Affiliations:** Department of Psychology, The University of Sheffield, Sheffield, UK; Universidad Autónoma de Chile, Chile; Department of Psychology, Edge Hill University, Ormskirk, UK

**Keywords:** sensory processing, learning, literacy, cerebellum, response selection, occupational therapy

## Abstract

There is pervasive evidence that problems in sensory processing occur across a range of developmental disorders, but their aetiology and clinical significance remain unclear. The present study investigated the relation between sensory processing and literacy skills in children with and without a background of special educational needs (SEN). Twenty-six children aged between 7 and 12 years old, from both regular classes and SEN programmes, participated. Following baseline tests of literacy, fine motor skills and naming speed, two sets of instruments were administered: the carer-assessed Child Sensory Profile-2 and a novel Audiovisual Animal Stroop (AVAS) test. The SEN group showed significantly higher ratings on three Child Sensory Profile-2 quadrants, together with body position ratings. The SEN participants also showed a specific deficit when required to ignore an accompanying incongruent auditory stimulus on the AVAS. Interestingly, AVAS performance correlated significantly with literacy scores and with the sensory profile scores. It is proposed that the children with SEN showed a specific deficit in “filtering out” irrelevant auditory input. The results highlight the importance of including analysis of sensory processes within theoretical and applied approaches to developmental differences and suggest promising new approaches to the understanding, assessment, and support of children with SEN.

Sensory processing abilities have been widely studied in a range of neurodevelopmental conditions. For example, the underlying sensory problems in autism spectrum disorders (ASDs) have been studied in both children and adults (Baker et al., 2008; Gonthier et al., 2016; Kwakye et al., 2011; Martínez-Sanchis, 2014; Tomchek et al., 2014). There is also evidence of sensory processing differences in people with attention deficit hyperactivity disorder (ADHD; Dunn & Bennett, 2002; Panagiotidi et al., 2017; Parush et al., 1997; Shimizu et al., 2014) and specific language impairment (McArthur & Bishop, 2005; Taal et al., 2013). In the case of specific reading disorder, also known as developmental dyslexia, phonological processing deficits are well established and represent the leading theory (Stanovich, 1988; Vellutino et al., 2004), which posits that reading and spelling difficulties result from impairments in phonological awareness and processing. Nonetheless, the underlying cause of the phonological problems remains unclear, and there are long-standing, albeit controversial, theories that claim that visual (Bouldoukian et al., 2002; Lovegrove et al., 1990; Stein & Walsh, 1997), auditory (Merzenich et al., 1996), and/or sensorimotor difficulties (Nicolson et al., 2001) are also significant causal issues. Recent formulations of these frameworks are provided in Stein (2019) for magnocellular visual and sensorimotor problems, in Nicolson and Fawcett (2019) for a general delay in developing “automatic” skills, and in Hancock et al. (2017) in terms of overall increased “neural noise” that leads to higher cortical excitability and diminished signal-to-noise ratio for sensory processing.

It is also well documented that reading disorders have high ‘comorbidity’ with other developmental disorders. The most common co-occurring difficulties are specific language impairment, ADHD, developmental coordination disorders, and dyscalculia. In addition, some people with dyslexia may experience traits of ASDs. The overlap among these disorders has been reported from 10% to more than 50% of the cases (Bental & Tirosh, 2007; Kirby et al., 2008; McArthur et al., 2000). This variety of characteristics reflects wider problems underlying dyslexia and involves an extra challenge for its understanding and intervention. Ironically, these comorbidity findings relate strongly to the conceptualisations of developmental disorders 40 years ago in terms of minimal brain dysfunction (Clements & Peters, 1962; Wender, 1978) or ‘soft neurological signs’ (Touwen & Sporrel, 1979).

Differences in sensory processing—the ability to register and modulate sensory information and to organise this sensory input to respond to situational demands (Humphry, 2002)—have also been widely studied in the context of occupational therapy (OT) for children. The profession of OT arose in the United States in response to the need for support for veterans of the First World War, both for physical and mental health. Jean Ayres (1963, 1972) was an early advocate of applying the OT approach to children with learning disabilities. Early OT work was on rehabilitation and recoordination of sensory, motor, and proprioceptive processing, and the concepts of vestibular function and “sensory integration,” deriving from acquired cases with wounded soldiers, were core to Ayres’ analysis and treatment approaches for the developmental cases on which she focused.

More recently, OT approaches to sensory processing have differentiated. Dunn’s model of sensory processing (Dunn, 1997) has been extensively used in research as an instrument to depict the sensory processing profile of children (Baker et al., 2008; Cheung & Siu, 2009; Dove & Dunn, 2008; Engel-Yeger & Dunn, 2011; Kern et al., 2007; Lowe et al., 2016; Padankatti, 2005; Taal et al., 2013; White et al., 2007). Dunn’s (1997) model posits that all of the senses—touch, smell, taste, sight, and sound, as well as physical movement and body awareness—are expected to have a “balanced” response to enable the adequate and adaptive functioning of brain mechanisms. According to Dunn (1997, 2001), the brain regulates the incoming messages and the consequent responses by a modulation process that provides a balance between the excitation and inhibition mechanisms regarding the available stimuli. An adequate sensory processing would occur only when internal (sensations of the body) and external (sensations of the environment) information are in a balanced state (Dunn, 2001).

Dunn’s (1997) framework is represented in a four-quadrant model that characterises the behaviours that people exhibit in their daily lives as a result of the interaction between two hypothetical constructs: neurological threshold (NT), the degree of stimulation required to activate sensory processing, and self-regulation strategies, the individual’s sensory regulation (SR) to adjust their sensory stimulation to their preferred range.

Dunn designed questionnaires for children and adults based on her model of sensory processing (Brown & Dunn, 2002; Dunn, 1997, 2014). These questionnaires create a profile that characterises the sensory preferences of the individual to sensory stimulation reflected by four “sensory quadrants”—Registration, Seeking, Avoiding, and Sensitivity—that represent the four possible combinations of NT and SR, with high scores for Registration indicating high NT, passive SR (i.e., they have low sensory sensitivity but do not actively seek sensory stimulation); high scores for Seeking indicating high NT, active SR (i.e., they have low sensory sensitivity and seek out higher levels of stimulation); high scores for Avoiding indicating low NT, active SR (i.e., they have high sensory sensitivity and actively avoid too much stimulation); and high scores for Sensitivity indicating low NT, passive SR (i.e., they have high sensory sensitivity but do not actively avoid sensory stimulation). Dunn (2007) provides clear examples of how parents can adjust a child’s environment to match their characteristic sensory processing quadrant (defined as the quadrant with the highest score).

Research has shown robust evidence of sensory issues in conditions such as ASD (Baker et al., 2008; Gonthier et al., 2016; Martínez-Sanchis, 2014; Tomchek et al., 2014) and ADHD (Dunn & Bennett, 2002; Parush et al., 1997). Furthermore, Dove and Dunn (2008) and Padankatti (2005) reported that children with special educational needs (SEN) presented a challenging profile of sensory processing, characterised by significant high scores in the quadrants of Seeking, Avoiding, and Registration. Unfortunately, there has been little research attempting to relate sensory profile research to underlying cognitive or cognitive neuroscience research. A recent study (Metz et al., 2019) on typically achieving (TA) adults failed to find linkage between Dunn’s hypothetical threshold measure and event-related potentials, but it is course likely that developmental sensory processing differences diminish by adulthood. Consequently, there is a clear need to probe the relationship between Dunn’s clinically relevant behavioural tests and established literacy and cognitive measures for children with SEN.

A recurring theme in OT work is the concept of sensory integration, and consequently, we wished to develop a test of audiovisual (AV) processing able to probe both AV integration and AV dissociation, and we therefore developed the AV Animal Stroop (AVAS) test, which allows these abilities to be assessed independently, as discussed later. The classic colour–word Stroop paradigm (Stroop, 1992) is a conflictual situation between a written colour name and the ink that it is printed (e.g., the word “blue” written in red). In this “response competition” situation, the naming of the colour of the ink is slowed significantly (with likely reduction in accuracy also). Although studies have shown lower performance in Stroop-like tasks in dyslexic readers compared with typical readers (Bucci et al., 2013; Faccioli et al., 2008), the classic Stroop approach exploits reading automaticity (in terms of the colour name word interfering with the ink colour sensory input), and this automaticity is less well-developed in poor readers, leading to difficulties in interpretation of any differences. Consequently, we wished to develop a version independent of reading and appropriate for use by young children with SEN.

## Method

### Participants

This study was approved by the ethical committee of The University of Sheffield, reference number 007264. The participants were 26 children aged 7 to 11 years old (13 females), and their parents from two primary schools in Sheffield, UK. One group comprised children who participated in SEN group (15 students), with the control group of TA children without a background of learning disorders. The groups included all children whose parents returned the ethical consent forms. The Children and Families Act 2014 stipulates that children will be incorporated into SEN support if they “have a learning difficulty or disability which calls for special educational provision to be made for them” (section 20 of the Act 2014, the website for the “UK Government Legislation,” can be found at http://www.legislation.gov.uk). It is important to note that the researchers did not have access to educational/clinical diagnosis of children in the SEN group, and, indeed, it is unlikely that those younger than 10 years of age had been assessed by an educational psychologist.

### Procedure

Members of the school staff referred children from either regular classes or the SEN programme. Through the school staff, letters were sent to the parents with an information sheet addressed to them and their child (in a child-friendly format), plus a consent form. Two hundred invitations to take part in the study were sent out, with a return rate of 13%. Parents who agreed to participate completed the Child Sensory Profile-2 (CSP-2; Dunn, 2014) questionnaire at home and then returned the form to school to be collected by the researcher. Children were tested with the Dyslexia Screening Test-Junior (DST-J; Fawcett & Nicolson, 2004) and the AVAS task on the school premises. The schools provided a quiet room for the testing, and it took a maximum duration of 30 minutes.

#### Assessment of Literacy Skills

The DST-J is designed to be administered by school professionals, takes about 30 minutes to be completed, and has been used for research purposes owing to its design for testing a spectrum of skills and attainments. The manual (Fawcett & Nicolson, 2004) gives mean test–retest reliability of the tests used here as 0.89. Interrater agreement (via video recording) between experienced testers was 0.98.

Five subtests of the DST-J were administrated to the participants in an individual one-test session during school time. The subtests selected and the order of presentation were as follows:

1. Rapid naming: a test of general linguistic fluency. It involves the time taken to speak the names of pictures on a page full of common objects. An established executive function test of speed of multi-modal processing based on the rapid automatised naming (RAN) test (Denckla & Rudel, 1976).
2. Bead threading: a test of fine motor skills. The test consists of seeing how many beads can be threaded in 30 seconds. A standard test of fine motor skills.
3. One-minute reading: a test of reading accuracy and fluency. The test measures the number of single words (in increasing difficulty) that can be read in 1 minute.
4. Phonemic segmentation and rhyming: a measure of phonological awareness. These test the child’s ability to play with the constituent sounds in words.
5. Two-minute spelling. This subtest assesses how many words the child can spell correctly in 2 minutes, while the tester dictates the words (in increasing difficulty).

The age-banded norms of the DST-J allow for conversion of the set of raw scores into an “at risk index”—one of five categories from “very strong risk” to “above average” (non-risk). The DST-J also allows deciles to be derived for each score with decile 1 corresponding to lowest 10% on the norms for that age. Deciles between 1 and 3 correspond to risk categories (1 = *high risk*, 2 = *moderate risk*, 3 = *mild risk*), and deciles over 4 correspond to normal or not-at-risk for the specific test (Fawcett & Nicolson, 2004).

#### Assessment of Sensory Processing Profile

The sensory processing profile of children was assessed with the CSP-2 (Dunn, 2014). The CSP-2 is a standardised questionnaire that measures sensory processing in children aged from 3 years to 14 years and 11 months. The questionnaire has demonstrated strong internal consistency (Cronbach’s α = .88 – .92) and test–retest reliability (*r* = .96 – .97). The questionnaire consists of 86 statements in 6 “sensory systems” categories (Auditory, Visual, Touch, Movement, Body Position, and Oral) that are Likert-style 5-point items, ranging from *almost never* to *almost always* (including a “does not apply” option) that elicit information about the child’s ways of responding to sensory experiences in everyday life. The statements are combined to give an overall score for each sensory system and then recombined to obtain the four sensory quadrants: Registration, Seeking, Sensitivity, and Avoiding. The quadrants and sensory systems are also classified into five categories using Dunn’s (2014) national normative data in terms of “clinical” risk levels.

Typically developing children are likely to present a balanced sensory profile with ratings located in the mean range. More extreme (higher or lower) ratings indicate abnormal sensory processing, with high scores across all the sensory quadrants reflecting a maladaptive behaviour to the environment (Dunn, 1997, 2014; Little et al., 2016).

#### The Animal Classification/Audiovisual Animal Stroop Task

The Animal Classification (AC)/AVAS task was designed by the authors to test the influence of a multi-sensory setting on the performance of participants. The sensory modalities were visual and auditory stimuli. The core task was based on an AC task representing a simpler version of the RAN task for naming simple pictures. RAN tasks are a good measure of cognitive skills linked with literacy (Nicolson & Fawcett, 1994; Nicolson et al., 2010; Tallal, 1980; Wolf & Bowers, 2000).

**Figure 1.**
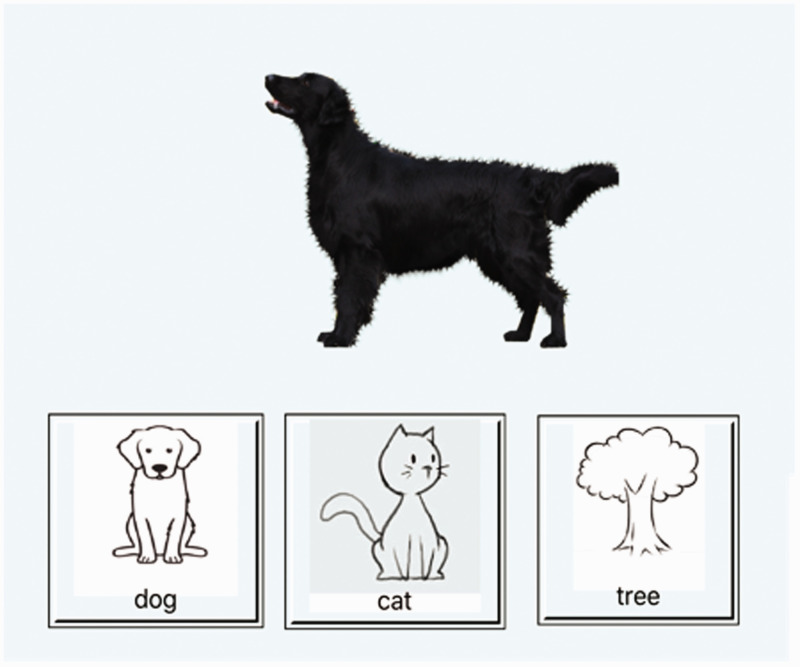
Sample Screen From AC/AVAS. For the standard version, only the stimulus picture was shown. For the AV condition, a compatible (bark for dog, mew for cat) or incompatible (bark for cat, mew for dog) noise was presented synchronously with the image onset.

In the present study, the AC/AVAS tasks were displayed on an iPad (model MNV62B/A, 9 inches) placed in front of the participants during all of the trials, at an approximate distance of 35 cm from their eyes. The complete AC/AVAS tasks were undertaken in two conditions of 1 minute each: the first condition was the (wholly visual) AC test, which involved timed classification of one of three types of “animal”—cat, dog, and tree—presented in a random order by the iPad. See Figure 1 for a sample picture, with the stimulus presented being the dog, and the user’s task is to tap the appropriate icon from the three at the bottom.

The second condition, which we have termed the AVAS task, was designed to probe sensory integration and sensory dissociation. It extended the unimodal AC tasks by presenting with the visual stimulus a sound associated with one of the target categories, creating a cross-modal situation. The sound was presented at the same time as the picture, randomly either as a congruent (e.g., an image of a dog and the sound of barking) or conflict (e.g., image of a dog and the sound of mewing) condition. For the picture of a tree, the accompanying sound was either mewing or barking. The sounds were played by the iPad’s speaker, which was set at the maximum volume. The performance on both conditions of the AC/AVAS task was rated on accuracy (the percentage of correct responses) and speed (the mean response time for correct responses).

## Data Analysis and Results

The analysis was carried out using parametric or non-parametric tests as appropriate following examination of the underlying response distributions. All statistical data analyses were performed using IBM SPSS Statistics for Windows, version 25.0. In cases where age norms were not available, the data were screened for age effects, but none were found, and so age is not explicitly included as a factor in the analyses.

### Literacy Skills

**Figure 2.**
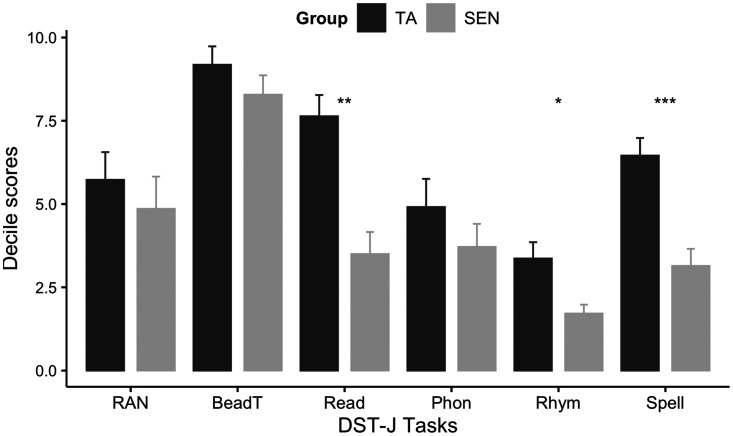
Dyslexia Screening Test-Junior. Significant differences between groups at ***< .001, **< .01, *< .05. Error bars represent standard error. RAN = rapid automatised naming; BeadT = bead threading; Read = reading; Phon = phonemic segmentation; Rhym = rhyming; Spell = spelling; TA = typically achieving; SEN = special educational needs; DST-J = Dyslexia Screening Test-Junior.

Performance on the DST-J subtasks (converted into age-normed decile scores) is shown in Figure 2. A Mann–Whitney test showed significant differences between the groups in Reading (*U* = 18.5, *p* =.001), Rhymes (*U* = 42.0, *p* = .046), and Spelling (*U* = 16, *p* < .001). The TA group obtained significantly higher scores (i.e., better performance) than the group of children with SEN. No significant differences between the groups were found in the rapid naming, bead threading, and phonemic segmentation tasks.

### Sensory Processing Profile

**Figure 3.**
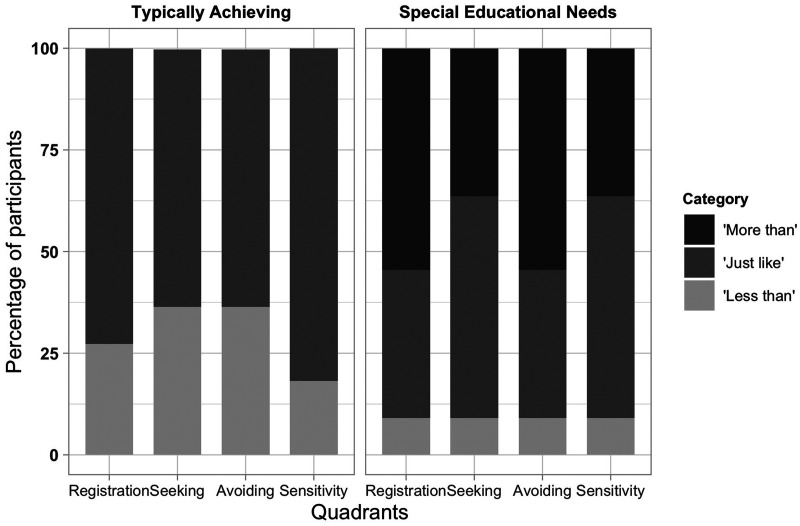
Representation of the Groups on Each of the Four Quadrants of Dunn’s Model Along the Categorical Ranges.

CSP-2 data for each participant for each “quadrant” were converted into an age-normed classification (less than others, just like the majority, more than others) using Dunn’s (2014) CSP-2 norms as shown in Figure 3. Somewhat surprisingly, no child in the TA group provided a rating in the “More than Others” category. By contrast, fewer children in the SEN group had ratings in the “Less than Others” category, with more (greater than 45%) in the “More than Others” category.

Mann–Whitney tests on the raw scores of the CSP-2 revealed significant between-group differences for the Registration, Avoiding, and Sensitivity quadrants after Bonferroni correction at the level of *p* < .0127. Use of Dunn’s (2014) national normative data indicated that Registration and Avoiding quadrants, but not the Sensitivity quadrant, demonstrated “clinical significance” according to Dunn’s scoring procedure (with scores at least 1 SD above the mean).

The analysis of the sensory systems showed significant differences in Auditory (*U* = 30.5, *p* = .028), Movement (*U* = 54.5, *p* = .045), and Body Position (*U* = 19.0, *p* = .004). After Bonferroni correction at *p* < .008 level, only the sensory system of body position remained statistically significant and also demonstrated “clinical significance.” Statistical analyses and normative ranges are shown in Table 1.

**Table 1.** Summary of the Scores on the CSP-2 Per Groups.

CSP-2	TA group	SEN group	Norms^a^	Mann–Whitney	Effect size
	*M* (*SD*)	*M* (*SD*)	*M* (*SD*)	*p*	*d*
Sensory quadrants					
Registration	22.00 (8.98)	44.00 (17.81)	31.4 (11.7)	.004	1.55
Seeking	22.45 (10.64)	38.27 (16.10)	35.9 (13.7)	.042	1.15
Avoiding	23.36 (6.93)	48.27 (18.11)	33.9 (12.5)	.002	1.81
Sensitivity	20.72 (8.22)	39.54 (20.18)	30.3 (11.0)	.006	1.22
Sensory systems					
Auditory	11.91 (6.13)	22.00 (10.20)	17.7 (6.9)	.028	1.19
Visual	8.91 (3.53)	11.83 (3.27)	13.6 (4.0)	.070	0.85
Touch	12.27 (7.32)	16.25 (10.13)	15.2 (6.9)	.477	0.45
Movement	7.82 (4.89)	14.83 (8.59)	13.7 (5.6)	.045	1.00
Body Position	6.18 (4.14)	15.17 (8.40)	10.0 (4.5)	.004	1.35
Oral	13.82 (9.91)	18.5 (14.00)	16.2 (7.4)	.518	0.38

*Note*. CSP-2 = Child Sensory Profile-2; TA = typically achieving; SEN = special educational needs.

^a^Norms correspond to the mean raw score extracted from the CSP-2 user’s manual (Dunn, 2014, p. 219). Effect size= Cohen’s *d*.

### AC and AVAS Performance

Two measures were derived from the AC and AVAS tasks: accuracy, which corresponded to the percentage of correct responses per trial, and reaction time, which is the average time of correct responses per trial in milliseconds. Mixed measures analysis of variance (ANOVA) was used to test the influence of Group and Condition for both measures. Inspection of the data for the accuracy scores indicated a non-normal distribution (with few errors), but parametric tests were chosen given that the Levene’s test of homogeneity was acceptable. Non-parametric tests provided equivalent findings for each condition.

### AC/AVAS Accuracy Scores

**Figure 4.**
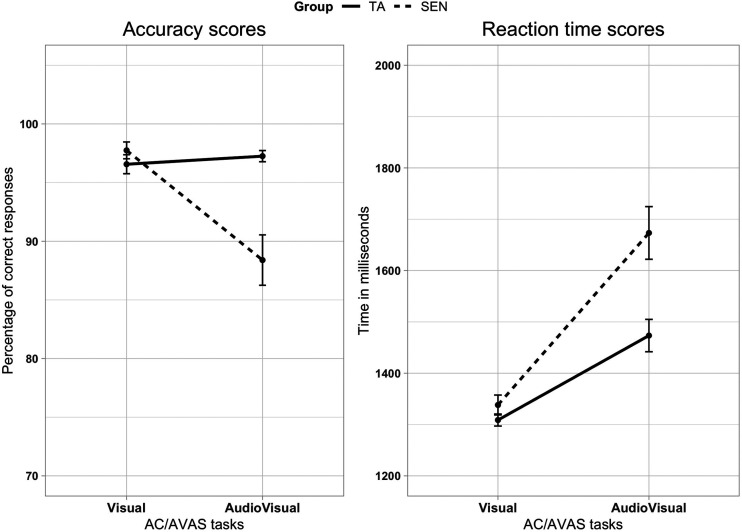
Line Plots Show Accuracy Scores and Reaction Time Scores by Both AC and AVAS Conditions. AVAS data include both congruent and conflict conditions. Error bars represent standard error. TA = typically achieving; SEN = special educational needs; AC/AVAS = Animal Classification/Audiovisual Animal Stroop.

A mixed 2 × 2 ANOVA was conducted with Group (TA, SEN) as the between-subjects variable and Condition (AC, AVAS) as the within-subjects variable. With homogeneity of variance assumed (Levenés test *p* > .05), there was a main effect of Condition, *F*(1, 22) = 6.19, *p* = .021, $\eta_{\text{ρ}}^{2}$ = .220, whereas that of Group was not significant, *F*(1, 22) = 3.72, *p* = .067, $\eta_{\text{ρ}}^{2}$ = .145. There was a significant interaction effect between Condition and Group, *F*(2, 44) = 8.30, *p* = .009, $\eta_{\text{ρ}}^{2}$ = .274. This arose because (only) in the AVAS condition, the SEN group were significantly less accurate (*M* = 88.39%, *SD* = 10.95) than the TA group (*M* = 97.24%, *SD* = 2.43, *p* = .016, $\eta_{\text{ρ}}^{2}$ = .238). By contrast, both groups performed equivalently in the AC condition. The interaction diagram for accuracy scores is shown in Figure 4.

For the AVAS condition, the effect of conflict/non-conflict of the auditory stimulus was not significant (*p* > .05).

### AC/AVAS Reaction Time Scores

For the AC/AVAS reaction time data, equivalent mixed 2 × 2 ANOVA was also undertaken, with group (TA, SEN) as the between-subjects variable and the two sensory conditions (AC, AVAS) as the within-subjects variable. With homogeneity of variance assumed (Levenés test *p* > .05), there was a main effect of Condition on the reaction time of participants, *F*(1, 22) = 46.02, *p* < .001, $\eta_{\text{ρ}}^{2}$ = .677, due to the participants being slower in the AVAS condition (*M* = 1573.33 ms, *SD* = 45.3) than the AC condition (*M* = 1323.40 ms, *SD* = 17.04). The Group variable did not show a significant effect on the reaction time, *F*(1, 22) = 3.93, *p* = .060, $\eta_{\text{ρ}}^{2}$ = .152. The interaction between the Group and Condition was also significant, *F*(1, 22) = 5.36, *p* =.030, $\eta_{\text{ρ}}^{2}$ = .196. Pairwise comparisons revealed that the significant effect was because the SEN group were slower than the control group in the AVAS condition (mean difference = 199.94, *p* = .38). Figure 4 also shows the interaction diagram for the reaction time scores.

**Figure 5.**
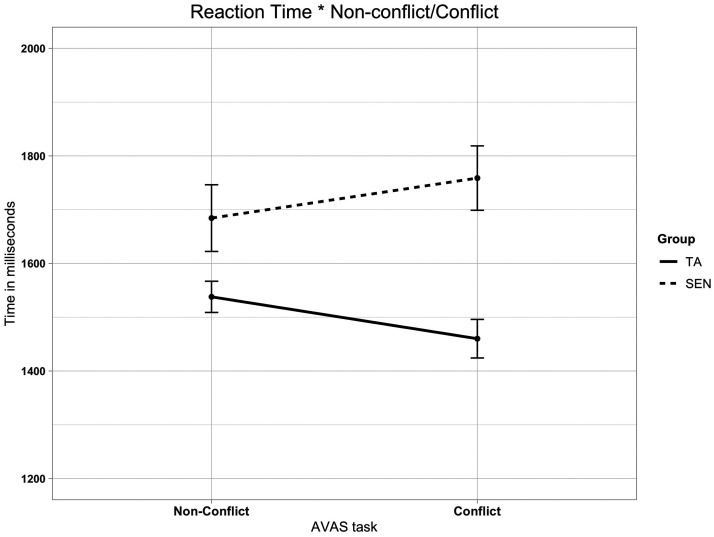
Line Plot Shows the Reaction Time Scores Interaction Between Conflict/Non-conflict Auditory Stimulus on the AVAS Condition. Error bars represent standard error. TA = typically achieving; SEN = special educational needs; AVAS = Audiovisual Animal Stroop.

Finally, the AVAS reaction time data were analysed separately to assess any interaction between Conflict (conflict vs. congruent) and Group (TA vs. SEN). For the accuracy data, there was a significant main effect of Group, *F*(1, 22) = 6.52, *p* =.018, $\eta_{\text{ρ}}^{2}$ = .229, but that of Conflict was not significant, *F*(1, 22) = 1.228, and the interaction was not significant, *F* < 1. For the reaction time data, there was a significant main effect of Group, *F*(1, 22) = 4.91, *p* =.037, $\eta_{\text{ρ}}^{2}$ = .182, but that of Conflict was not significant, *F*(1, 22) = 0.003, whereas the interaction was significant, *F*(1, 22) = 6.76, *p* =.016, $\eta_{\text{ρ}}^{2}$ = .235. The pairwise comparison showed that the interaction effect was due to the SEN group (*M* = 1758.7 ms, *SD* = 305.3) being significantly slower on the conflict condition than the TA group (*M* = 1460.0 ms, *SD* = 183.0). See results in Figure 5.

### Association Between Literacy Tasks and Sensory Measures

A Spearman rank correlation test was used to assess the relationship among literacy skills (DST-J), AC/AVAS performance, and the scores of the sensory processing profile (CSP-2). See Table 2 for the full set of intercorrelations.

**Table 2.** Intercorrelations Between Measures of the DST-J, AC/AVAS, and CSP-2.

	1	2	3	4	5	6	7	8	9	10	11	12	13	14	15	16	17	18	19
DST-J																			
1. RAN																			
2. BeadT	–.063																		
3. Read	.556**	.396*																	
4. Phon	.314	.127	.531**																
5. Rhym	.409*	–.166	.393	.217															
6. Spell	.356	.232	.761**	.414*	.456*														
Animal classification																			
7. AC Acc	.567**	–.389	.080	.257	.112	.089													
8. AC RT	–.231	.164	–.389	–.093	–.291	–.527**	–.302												
9. AVAS Acc	.156	.059	.419*	.270	.444*	.671**	.244	–.514*											
10. AVAS RT	–.472*	.088	–.505*	–.118	–.228	–.525**	–.303	.732**	–.586**										
CSP-2																			
11. Registration	–.195	–.183	–.351	–.319	–.131	–.232	–.014	–.249	–.575**	.139									
12. Seeking	.021	–.075	–.070	–.350	–.073	–.254	–.204	–.074	–.672**	.020	.766**								
13. Avoiding	–.209	–.079	–.459*	–.278	–.139	–.385	–.213	–.017	–.641**	.346	.877**	.622**							
14. Sensitivity	–.123	–.057	–.218	–.278	–.239	–.283	–.066	–.228	–.636**	.111	.919**	.834**	.862**						
15. Auditory	–.081	.093	–.161	–.078	–.437*	–.245	–.061	.149	–.542*	.123	.646**	.581**	.747**	.760**					
16. Visual	–.174	–.234	–.192	–.136	–.027	–.191	–.071	–.207	–.317	–.106	.641**	.513*	.623**	.577**	.605**				
17. Touch	.009	.086	.061	–.035	–.073	.028	–.107	–.296	–.346	–.013	.707**	.698**	.565**	.714**	.400	.229			
18. Movement	.005	.070	–.079	–.274	–.077	–.263	–.113	–.083	–.579**	.017	.763**	.940**	.563**	.801**	.434*	.410	.695**		
19. Body Position	–.208	–.324	–.422	–.154	.050	–.213	.060	–.352	–.316	.023	.843**	.493*	.781**	.704**	.332	.422*	.475*	.531**	
20. Oral	–.068	0	.014	–.344	–.008	–.172	–.378	–.169	–.404	.034	.570**	.753**	.491*	.626**	.342	.228	.781**	.755*	.375

*Note*. DST-J = Dyslexia Screening Test-Junior; RAN = rapid automatised naming; BeadT = bead threading; Read: 1-minute reading; Phon = phonemic segmentation; Rhym = rhymes; Spell = spelling; AC = unimodal condition (visual stim); AVAS = cross-modal condition (audiovisual stim); Acc = accuracy scores; RT = reaction time scores; CSP-2: Child Sensory Profile-2 questionnaire: quadrants and sensory systems.

*p < .05. **p < .01.

For DST-J and AC/AVAS measures, there were significant correlations among (from more to less number of significant correlations) spelling, RAN, reading, and rhyming subtests with some of the AC/AVAS scores. These findings demonstrate an association between the AVAS performance and literacy skills.

For CSP-2, there were highly significant positive intercorrelations between the majority of the measures. For DST-J and CSP-2, there were few significant correlations. Only a negative correlation with medium effect was found between the quadrant of Avoiding and Reading (*r* = –.459, *p* = .036) and between the sensory system of Auditory and the Rhyming subtest (*r* = –.437, *p* = .042).

Finally, the analyses between the CSP-2 and AC/AVAS measures showed a significant negative correlation between the accuracy scores in the AVAS condition and all the four sensory quadrants and the auditory and movement sensory systems.

## Discussion

The study was designed to gather rich data on sensory processing in children with SEN and to relate these findings to more traditional cognitive performance tests. The tests used were five subtests of the DST-J (Fawcett & Nicolson, 2004), the CSP questionnaire (Dunn, 2014), and a custom-designed AC task to assess sensorimotor integration and dissociation. We take the results in turn.

On the DST-J, as expected, there were significant and substantial between-group differences on the literacy measures for reading, spelling, and rhyme. Performance on bead threading, rapid naming, and phonological processing was also lower, but not significantly so, for the SEN group.

For the sensory processing profile data, the SEN group ratings were higher on all four quadrants, with significantly higher ratings for Registration, Avoiding, and Sensitivity, but only Registration and Avoiding reached Dunn’s criterion for clinical significance. For the sensory system ratings, the SEN group had markedly higher ratings on the Auditory, Movement, and Body Position measures, with only the latter surviving the Bonferroni correction.

For the AC/AVAS task, no between-group difference was found for the AC condition. However, a significant interaction was found both for the accuracy and the response speed data when AC was compared with AVAS. The SEN group were markedly impaired on both speed and accuracy for the AVAS condition, whereas the TA group showed only modest decrements, with the major between-group effect deriving from the conflict stimuli within AVAS.

Finally, the correlational analyses revealed high intercorrelations for almost all the sensory profile and sensory processing ratings. Furthermore, AVAS accuracy correlated significantly with DST-J Reading, DST-J Rhyme, DST-J Spelling, and with all four CSP-2 Quadrants, together with the Auditory and Body Position sensory processing ratings.

The high scores on the CSP-2 ratings are consistent with previous studies (Dove & Dunn, 2008; Padankatti, 2005) that compared children with learning disorders with typically learning children. The manual of the CSP-2 (Dunn, 2014) states that high scores on Registration indicates that the child “may miss sensory input needed for participation”; while high scores on Sensitivity indicate that the child “may be so distracted by sensory input that it interferes with participation”; and high scores on Avoiding indicate that the child “may become overwhelmed by stimuli, thus actively would try to avoid them.”

The results from the present study (in common with results of earlier studies) appear to present a challenge to Dunn’s NT/SR framework. In that the SEN group ratings indicated clinically relevant high scores both for Registration and Avoiding. Dunn claims that high scores on Registration reflect high NT, low SR, whereas high scores for Avoiding represent low NT, high SR. This is precisely the opposite configuration, thereby contradicting any possible prediction of the framework. While Dunn’s NT/SR framework (Dunn, 1997) does propose that apparently inconsistent quadrants can coexist in the same child, this contradiction does seem to suggest that the quadrant framework is not easily testable and hence that it is not capturing the right classificatory dimensions.

In terms of the sensory processing profile, the major between-group difference was for Body Position, an index of proprioceptive sensitivity with representative probes being “Seems to have weak muscles” and “Walks loudly as if feet are heavy.” Movement Processing (e.g., “Loses balance unexpectedly when walking on an uneven surface,” “Bumps into things, failing to notice objects or people in the way”) and Auditory Processing (e.g., “Is distracted when there is a lot of noise around,” “Tunes me out or seems to ignore me”) also approached significance.

The Body Position index appears to be closely aligned with cerebellar function, in that low muscle tone and suboptimal motor coordination are its classic signs (Holmes, 1939) as are the two Movement Processing questions noted here. The Auditory Processing questions appear at first sight somewhat contradictory, indicating both higher and lower thresholds, analogous to our findings with the quadrants.

The AVAS data provide a strong steer towards an explanation of this apparent contradiction. The data indicate that the SEN group were less efficient at filtering out stimuli on a “sometimes-to-be-ignored” auditory dimension, which is directly consistent with the “is distracted by noise” question. By contrast, the TA group were able to filter out the conflicting auditory stimuli with little decrement to accuracy or speed. These results indicate that the TA group had available an effective sensory filtering system and were, therefore, able to undertake the task “automatically,” whereas the SEN group did not and, consequently, had to undertake “controlled processing” (Shiffrin & Schneider, 1977) to complete the task.

Therefore, if one includes both “threshold” and “processing mode” (controlled versus automatic), it is possible to provide a coherent interpretation of all the results. In particular, we propose (in agreement with Dunn) that the SEN children tend to have a higher NT and also (in contrast to Dunn) less efficient “sensory filtering,” that is, focusing on one sense and excluding the others (Broadbent, 1958). In circumstances where “auditory noise” is affecting their concentration, they will avoid the situation, whereas in situations where senses combine helpfully, they will prefer higher intensity sensory input.

This interpretation is supported by the strong intercorrelations between AVAS accuracy scores and all the sensory profile ratings. Furthermore, the significant intercorrelations with DST-J literacy scores suggest that the AVAS task may tap an important underpinning skill for literacy. The AC (visual only) speed score correlates only with the DST-J spelling score. By contrast, the AVAS speed score does not intercorrelate with the sensory profile data but does correlate with four DST-J tests (rapid naming, reading, rhyme, and spelling), indicating that auditory–visual conflict does tap an important dimension of the processes of learning to read.

Before considering potential theoretical interpretations of these results, it is important to acknowledge the clear limitations of this study. The primary limitation is of course the small sample size both for TA and for SEN children, compounded by the relatively wide age range involved. This strongly limits the generality of the results obtained and highlights the need for further attempted replications. It should be stressed, however, that the samples taken were the full set of those children (and parents) who agreed to participate using the ethics participation request, and so these children were representative of the schools involved.

The secondary limitation, or perhaps feature, of these data, is that there was no information provided by the schools about the SEN participants, except that they were in their SEN support systems. Indeed, the schools themselves had no externally validated diagnostic information available owing to the lengthy nature of SEN diagnosis in the UK schools. In principle, these children might be diagnosable with any (or several) of a range of specific learning difficulties from dyslexia to ADHD to language disorder to ASD. The robust nature of the differences found, especially given the relatively low numbers involved, is therefore particularly noteworthy and highlights the value of avoiding premature specificity in participant selection given the major overlap between the symptoms of many learning disorders (Gilger & Kaplan, 2001; Kadesjo & Gillberg, 2001; Landerl & Moll, 2010; Nicolson & Fawcett, 2007).

Turning to theoretical interpretations, the finding of a higher sensory threshold is consistent with broader frameworks for dyslexia, including the recent “neural noise” hypothesis (Hancock et al., 2017) and the “delayed neural commitment” framework (Nicolson & Fawcett, 2019). By contrast, the specific impairment of the SEN group in the conflict condition of the AVAS is a novel finding with strong theoretical implications. In particular, it highlights the fact that “sensory selection” can be at least as important as “sensory integration.” Current evidence (Dionne-Dostie et al., 2015) indicates that routine sensory integration is acquired within the first year of life, though the ability does continue to develop, with the superior colliculus playing a central role (Stein et al., 2009). By contrast, the developmental literature on sensory selection—consciously focusing on one sensory dimension rather than another—is sparse but is generally considered as an aspect of executive function that develops through childhood (Diamond, 2013), with considerable individual differences (Barutchu et al., 2019; Hirst et al., 2019).

The AC/AVAS and sensory profile findings provide strong constraints on potential interpretations. It is now accepted that integration of sensory and proprioceptive data takes place in the cerebellum (Herzfeld & Shadmehr, 2014; Manzoni, 2005; Marr, 1969), that the cerebellum plays a key role in sensory integration disorders (Koziol et al., 2011), and that the cerebellum may act as an “adaptive filter” to undertake—adaptively—a linear combination of the different sources of sensory input to it (Porrill et al., 2013; Raymond & Medina, 2018). Consequently, it is likely that the circuitry for learning to “filter out” the auditory modality does include the cerebellum, together with other subcortical structures.

It is notable that abnormal function in subcortical networks—especially those involving the cerebellum—has been implicated in most developmental disorders including ADHD, dyslexia, and ASD (Nicolson & Fawcett, 2007; Stoodley, 2016). It is also of interest to our hypothesis that the way the AVAS task forces the SEN group to “consciously compensate” for a lack of automaticity in sensory filtering is predicted by the Dyslexia Automatisation Deficit framework (Nicolson & Fawcett, 1990), which was subsequently integrated into the Cerebellar Deficit framework (Nicolson et al., 2001).

It is also important to note that sensory processing difficulties in vision or audition have been a long-standing component of dyslexia theories from the phonological deficit framework (Stanovich, 1988; Vellutino et al., 2004) to the magnocellular deficit frameworks (Stein, 2001; Tallal et al., 1993). Vellutino’s (1979) seminal research building on Liberman’s language-based approach to dyslexia (Liberman et al., 1971) actually highlighted a between-modality problem in visual-verbal learning, a concept further developed by Blau et al. (2009).

It must also be noted that combining Dunn’s OT sensory integration approach with executive function tests based on current models of attentional networks and their development (Posner et al., 2016) has provided results that link the disciplines and could prove the basis for fruitful further research, integrating theory, assessment, and intervention for children in and out of school.

In particular, use of the AC/AVAS appears in itself to give a simple assessment of visual executive function (via AC) and of the function of the executive control system and of the automaticity of auditory filtering for the child in question. If there is a limitation in auditory filtering, it is certainly appropriate to adjust the child’s home environment to make it more congenial—as reflected in Dunn’s suggestions to parents (Dunn, 2007)—but this is also important within the school environment, where noisy classrooms have become the norm. Furthermore, a pressing need in terms of intervention would be to help the child develop the necessary auditory filtering capability, perhaps through some appropriately configured game format.

In terms of theory, there were significant correlations between auditory filtering capability and literacy (reading, spelling, and RAN but not phonological processing), and sensory systems (Auditory—e.g., “My child struggles to complete tasks when music or TV is on”—and Movement—e.g., “My child takes movement or climbing risks that are unsafe.”). It is evident that the linkage between these capabilities is indirect, a finding that supports the OT approach of looking for sensory-motor-vestibular maturational processes as underlying causes, although it does not support the traditional OT focus on sensory integration (Ayres, 1972), rather the opposite in terms of sensory dissociation. In terms of modern cognitive neuroscience, it supports the general framework of development of functional connectivity networks (Buckner et al., 2011; Marusak et al., 2017; Yeo et al., 2011).

The performance of children with SEN on sensory profile questionnaires revealed highly significant differences in “behavioural sensory threshold,” especially for “Body Position.” Furthermore, performance on the newly developed AVAS test revealed that the children with SEN had impaired ability to filter out the auditory channel when undertaking a visual task. The results are interpreted in terms of impaired automatic sensory selection ability, leading to the need to consciously compensate to achieve adequate performance. This dual process explanation also provides a coherent explanation for the sensory profile findings.

In conclusion, we believe that use of the AVAS test can prove a fruitful tool for studies of sensory processing, and in particular, the findings highlight the importance of including analysis of sensory processes in the understanding, assessment, and support of children with SEN.
